# Longitudinal associations between structural prefrontal cortex and nucleus accumbens development and daily identity formation processes across adolescence

**DOI:** 10.1016/j.dcn.2020.100880

**Published:** 2020-11-11

**Authors:** Andrik I. Becht, Eduard T. Klapwijk, Lara M. Wierenga, Renske van der Cruijsen, Jochem Spaans, Laura van der Aar, Sabine Peters, Susan Branje, Wim Meeus, Eveline A. Crone

**Affiliations:** a Erasmus School of Social and Behavioural Sciences, Erasmus University Rotterdam, The Netherlands; b Research Center Adolescent Development, Utrecht University, The Netherlands; c Brain and Development Research Center, Leiden University, The Netherlands

**Keywords:** Structural brain development, Adolescent identity formation, Medial PFC, Lateral PFC/ACC, Nucleus accumbens, Longitudinal

## Abstract

We tested whether adolescents with daily high identity uncertainty showed differential structural brain development across adolescence and young adulthood. Participants (*N* = 150, *M*_ageT1_ 15.92 years) were followed across three waves, covering 4 years. Self-reported daily educational identity and structural brain data of lateral prefrontal cortex (lPFC)/anterior cingulate cortex (ACC), medial PFC, and nucleus accumbens (NAcc) was collected across three waves. All hypotheses were pre-registered. Latent class growth analyses confirmed 2 identity subgroups: an identity synthesis class (characterized by strong commitments, and low uncertainty), and an identity moratorium class (high daily identity uncertainty). Latent growth curve models revealed, on average, delayed maturation of the lateral PFC/ACC and medial PFC and stable NAcc. Yet, adolescents in identity moratorium showed lower levels and less decline in NAcc gray matter volume. Lateral PFC/ACC and medial PFC trajectories did not differ between identity subgroups. Exploratory analyses revealed that adolescents with higher baseline levels and delayed maturation of lateral PFC/ACC and medial PFC gray matter volume, surface area, and cortical thickness reported higher baseline levels and stronger increases of in-depth exploration. These results provide insight into how individual differences in brain development relate to fluctuations in educational identity development across adolescence and young adulthood.

## Highlights

• We tested associations between structural brain development and identity.
• We found 2 identity classes: an identity synthesis and identity moratorium class.
• LPFC/ACC and MPFC and nucleus accumbens showed delayed maturation in adolescence.
• Identity subroups differed on nucleus accumbens level and change.
• Delayed maturation of LPFC/ACC and MPFC predicted in-depth identity exploration.

## 1 Introduction

One of the key tasks for adolescents is to find out who they are as a person, also referred to as the process of developing a personal identity (e.g., Erikson, 1968; Marcia, 1966; Meeus et al., 2010). Identity development is defined as a search for self-defined values and commitments in various life domains, such as the educational and occupational domain and commitments to relevant others (Crocetti et al., 2008; Goossens, 2001). Two key processes are involved in the process of identity formation. *Commitment* refers to strong choices that adolescents have made in various life domains. *Reconsideration of commitment* represents individuals’ identity uncertainty and willingness to discard commitments and search for new ones (e.g., Crocetti et al., 2008; Meeus et al., 2010). Being unable to develop strong identity commitments can be very stressful for adolescents. Prior research revealed, for instance, that ongoing identity uncertainty (i.e., continuous reconsideration of commitments) predicts increasing depressive symptoms during adolescence (Becht et al., 2019) and is a risk factor for developing a range of other psychosocial adjustment problems (for reviews see Meeus, 2011; Van Doeselaar et al., 2018). In the present study we therefore examine longitudinal structural brain development patterns to test whether the subgroup of adolescents who experience daily identity uncertainty (Becht, Nelemans, et al., 2016) differ in their structural brain development compared to adolescents who establish stable and strong identity commitments. All hypotheses of the present study were pre-registered here https://osf.io/np5hz/.

### 1.1 Neurobiological correlates of identity development

There is a large interest in how individual differences in brain development relate to individual differences in psychological outcomes (Rosenberg et al., 2018). Recent scholars highlight the importance to study how variability in brain development is associated with variability in behavioral development, including identity formation (Becht and Mills, 2020; Foulkes and Blakemore, 2018). Prior longitudinal studies on structural brain development in other behavioral processes revealed that individuals with stable high levels of prosocial behavior showed greater cortical thinning in social brain areas (mPFC, TPJ, and STS; Ferschmann et al., 2019). Similarly, individuals with a more mature personality profile (indicated by higher levels of conscientiousness, emotional stability and imagination) showed a relatively faster rate of cortical thinning in adolescence that slowed to less thinning during the transition to young adulthood (Ferschmann et al., 2018).

Prior work on identity development specifically showed that individual differences in structural brain development of the lateral prefrontal cortex and ventral striatum predicted individual differences in adolescents’ identity (Becht et al., 2018). Specifically, higher levels of nucleus accumbens (NAcc) gray matter volume predicted higher commitment levels and lower reconsideration levels. Higher levels and delayed maturation (i.e., a less steep decline) of lateral prefrontal cortex gray matter volume predicted more in-depth exploration. These findings were interpreted to suggest that the prefrontal cortex and ventral striatum, previously found to be important for pursuing self-defined goals (Crone and Fuligni, 2020; Tamnes et al., 2017) and motivation and affect (Urosević, Collins, Muetzel, Lim, & Luciana, 2012), contribute to identity development as well.

The goal of the current study was to replicate and extend these previously obtained results on the associations between structural development of lateral PFC/ACC and the NAcc and identity development. We tested these associations in a new developmental longitudinal study (the Leiden Self-Concept study). The previous study by Becht et al. (2018) focused on the interrelations between identity, the NAcc, and the lateral PFC/ACC regions of interest (Mills et al., 2014a,b). Here, we extend this selection with the medial PFC (mPFC), given that this region is considered not only an important part of the social brain network (Mills et al., 2014b), but is also particularly involved in self-referential processes (Crone and Fuligni, 2020; Denny et al., 2012). For example, functional imaging studies indicate that the mPFC is consistently recruited when adolescents think about themselves (compared to descriptions of traits of others or traits in general; Denny et al., 2012; Pfeifer et al., 2013; van der Cruijsen et al., 2018; Van der Cruijsen et al., 2019), and when adolescents think about their past, present and future self (Pfeifer and Peake, 2012). Given that the mPFC continues to develop during adolescence (Mills et al., 2014b), a critical question remains whether individual differences in structural development of the mPFC may be associated with identity formation processes as well.

### 1.2 Individual differences in (daily) identity formation processes across development

Contemporary theoretical and empirical studies into adolescents’ identity highlight that identity formation is best described by two interrelated processes of commitment making and reconsideration of alternative identity commitments (Crocetti et al., 2008; Luyckx et al., 2006; Marcia, 1966). Identity development is considered a dynamic self-organizing process that fluctuates from day to day (Bosma and Kunnen, 2001; Lichtwarck-Aschoff et al., 2008; van der Gaag et al., 2016). As such, daily assessments can provide us with information on identity fluctuations and thereby provide a richer and more reliable picture of adolescents’ identity development (Becht, Branje, et al., 2016; Crone and Fuligni, 2020). For instance, daily identity commitment and reconsideration processes mutually affect each other in early adolescence (Klimstra et al., 2010). Besides, increasing commitment fluctuations (but not commitment levels) predicted less reconsideration across adolescence (Becht, Nelemans, Branje et al., 2017). Together these findings highlight that identity formation processes operate across days and daily identity measures capture potentially meaningful variance.

This study therefore examined identity development as a compound score across one-week daily assessment that were collected in a 3-annual longitudinal study (the Leiden Self-Concept study; van der Cruijsen et al., 2018). While previous daily diary studies revealed that identity formation processes operate on a daily basis, the patterns of these processes may differ across adolescents. Longitudinal research on daily identity processes indeed discovered that a relatively large subgroup of adolescents (i.e., around 50% of the total sample) experienced the often described pattern of an identity crisis (Becht, Nelemans, et al., 2016), or identity moratorium (Marcia, 1966), with higher levels and a peak in identity reconsideration during middle adolescence (ages 15–17 years), higher commitment fluctuations and weaker commitments across days compared to adolescents in the identity synthesis class (Becht, Nelemans, et al., 2016). Adolescents in the identity synthesis class (often referred to as the identity achievement status, Marcia, 1966) showed a pattern of identity maturation as identified in earlier work (for reviews see Kroger et al., 2010; Meeus, 2011, 2018), including lower levels of identity reconsideration, less commitment fluctuations and stable high commitment levels. Here, we aimed to replicate these two patterns of identity subgroups (2016), and we examine whether these subgroups show differential patterns of structural brain development (Becht and Mills, 2020; Foulkes and Blakemore, 2018).

### 1.3 Overview of the present study

The main objective of the present pre-registered study was to examine whether adolescents with high daily levels of identity uncertainty differ in the developmental trajectories of structural brain development in the lateral PFC/ACC, the medial PFC and NAcc compared to adolescents with higher identity certainty. All our hypotheses on the number and developmental shape of the identity classes, structural brain development and our hypotheses on the linkages between brain development and identity were pre-registered here: https://osf.io/np5hz/. In short, we expected to find two identity trajectory classes; one identity uncertainty class, and one identity synthesis class (Becht, Nelemans, et al., 2016).

Second, we predicted non-linear decreasing lateral PFC/ACC and medial PFC volume and surface area across adolescence, with stable levels in early adolescence (10–13 years) and decreasing levels from middle adolescence into young adulthood (15–25 years) (Becht et al., 2018; Mills et al., 2014a,b; Tamnes et al., 2017), and a linear decrease of lateral PFC/ACC and medial PFC thickness across adolescence (Wierenga, Langen, Oranje et al., 2014). For NAcc we predicted stable levels of NAcc volume over time (Becht et al., 2018; Herting et al., 2018).

Third, we predicted that adolescents in a trajectory class that is characterized by high daily identity uncertainty show relatively lower levels of lateral PFC/ACC and medial PFC volume, surface area and cortical thickness and lower levels of NAcc volume (Becht et al., 2018). Besides, based on a window of opportunity principle of brain development (Crone and Dahl, 2012), we expect that delayed brain maturation in relevant brain regions signals increased plasticity for adolescent to develop a strong identity. This increased plasticity may provide adolescents with opportunities to learn new skills but also to explore their identity (Blakemore & Mills, 2014). Based on this developmental principle, we predicted that those adolescents in the high daily identity uncertainty trajectory class show faster decreasing levels in lateral PFC/ACC and medial PFC volume, surface area and thickness, and decreasing levels of NAcc volume relative to adolescents in a high identity certainty (i.e., identity synthesis class) (Becht et al., 2018). For reasons of model complexity, we did not examine differences in non-linear quadratic slopes between identity subgroups.

In the first confirmatory section we tested the pre-registered hypotheses. In addition, we conducted non-pre-registered exploratory analyses on the longitudinal associations between in-depth exploration and lateral PFC/ACC and medial PFC, as in-depth exploration was previously predicted by the lateral PFC/ACC (Becht et al., 2018), but was not part of our pre-registered analyses. In-depth exploration captures individual’s continuous monitoring of their present commitments, by reflecting on their choices and search for information about these commitments in order to further strengthen and maintain these commitments (e.g., Crocetti et al., 2008; Meeus et al., 2010).

## 2 Method

### 2.1 Participants and procedure

Participants of this study included 160 right-handed Dutch adolescents at T1 (54 % girls; *M_age_* T1 = 15.92 years, *SD* = 2.97 range T1 = 11.00–21.17 years) who participated in the ongoing accelerated longitudinal Leiden Self-Concept study (for an elaborate description of the sample and procedure see van der Cruijsen et al., 2018). This longitudinal study includes three assessment waves (T1-T3) that were separated by a 1-year-and-3-months interval. In order to get a balanced number of participants in each age category across waves, we included N = 15 additional participants in the younger age range (around 10 years of age) at T2 and additional N = 14 older participants (i.e., around 23 years of age) at T3. As a result, the final sample included 198 participants. Figure S1 shows that the number of participants in each age group was balanced.

When participants came to the lab for the scan session, they were instructed to lie as still as possible during the whole scan period. Participants could watch a movie of their own choice during the high-resolution scan, which was administrated at the end of the scan session. Participants older than 18 years received €50 (equivalent to ∼ US$54) for participation at each assessment wave. Participants younger than 18 years received €40 (equivalent to ∼ US$44). Over the course of 4 years, adolescents participated in 3 assessment weeks. In each assessment week, participants filled out an online questionnaire tapping into their identity formation processes for five days in a row, resulting in 15 possible assessment days for each individual. The online assessments started 1-day after participants visited the lab for the scan session. Written informed consent was obtained from all participants and both parents of minors before inclusion in the study. The study was approved by the University Medical Ethical Committee. Before the scan session, participants were screened for MRI contra-indications and self-reported psychiatric disorders.

### 2.2 Daily identity formation processes

Daily identity commitment, reconsideration and in-depth exploration was assessed with the single-item version of the Utrecht Management of Identity Commitments Scale (U-MICS; Becht, Branje, et al., 2016; Klimstra et al., 2010). Single-items were directly derived from the original full 26-item original U-MICS questionnaire (Crocetti et al., 2008; Meeus et al., 2010). Confirmatory factor analyses (CFAs) on this full U-MICS questionnaire revealed that the commitment question “My school gives me certainty in life” and the reconsideration question “In fact, I am looking for a different school) had high factor loadings on the respective latent commitment and reconsideration factors (with factor loadings ranging between .86 and .95 across four waves (See Becht, Nelemans, Branje et al., 2017; for details about the factor analyses and results). The slightly adapted single item for commitment was “Today I felt confident about myself because of my school/study/work”. The item for identity reconsideration was “Today I felt that I could better look for a different school/study/work”. For our exploratory analyses we used the single item of in-depth exploration which is “Today I often thought about my school/study/work”. Items were rated on a 5-point likert scale (1 = *completely untrue*, 5 = *completely true*). Participants were asked to indicate whether they filled out the identity questions when thinking about their school/study or work. The majority of adolescents (74%) did not think about work when answering the identity questions. Only one adolescent reported in 8 out of 15 days to think about the work domain when answering the identity questions. Therefore, we recoded the scores on work identity into missing values and focused on the educational (i.e., school/study) identity domain. Validity and reliability of the single-item U-MICS questions for commitment, reconsideration and in-depth exploration have been supported (Becht, Branje, et al., 2016; Klimstra et al., 2010). Longitudinal measurement invariance of these items has been supported in adolescence across ages 13–18 years (Becht, Branje, et al., 2016). Moreover, the full 26-item U-MICS questionnaire from which these single items were derived shows longitudinal measurement invariance from early adolescence into emerging adulthood (ages 10–25 years) as well (Crocetti et al., 2015, 2008; Morsunbul et al., 2014). These findings further support the use of these identity questions in our sample with a similarly broad age range. Besides, several longitudinal studies modelled theoretically meaningful identity status trajectory classes from early to late adolescence (12–20 years; Meeus et al., 2010, 2012), and into young adulthood (10–29 years; Crocetti et al., 2012) based on the U-MICS questionnaire.

Factorial validity of the full U-MICS questionnaire has been supported in more than 10 different countries (e.g., Crocetti et al., 2015). Moreover, the U-MICS identity dimensions have been meaningfully related to other identity measures, such as the Ego Identity Process Questionnaire-Short form (Zimmermann et al., 2012). Validity of the single-item U-MICS has been supported as well (for an extensive discussion see Becht, Nelemans, Branje et al., 2017). For instance, based on adolescents’ scores on the single-item commitment and reconsideration questions it was possible to reliably classify adolescents in theoretically meaningful identity statuses (Becht, Nelemans et al., 2016). Besides, individual differences on these single-item commitment and reconsideration scores predicted internalizing and externalizing problem behaviors (Becht, Nelemans, et al., 2016; Schwartz et al., 2012), and academic adjustment (Klimstra et al., 2010).

For our subsequent analyses we computed a weekly mean level score and a weekly fluctuation score for educational identity commitment. Fluctuation scores were based on adolescents’ within-person standard deviation score per week (Kernis et al., 1989; Klimstra et al., 2010). Similar to, Becht, Nelemans et al. (2016) reconsideration level and fluctuation scores were highly correlated (ranging between 0.52 and 0.65 in our study). We therefore standardized and summed the reconsideration level and reconsideration fluctuation scores and used this combined score into the LCGA analyses, consistent with the study by Becht, Nelemans et al. (2016). Thus, we created one commitment level, one commitment fluctuation and one reconsideration score.

### 2.3 Neuroimaging

All participants were scanned on the same 3 T MRI scanner (Tesla, Philips Achieva MRI system Best, The Netherlands). The same procedure was uses as described in Becht et al. (2018). Tesla Philips Achieva MRI system, with a standard whole-head coil (Philips, Best, The Netherlands) at Leiden University Medical Centre. High-resolution T1-weighted anatomical scans were acquired (TR = 9.8 ms, TE =4.6 ms, flip angle = 8°, 140 slices, 0.875 mm x 0.875 mm x 1.2 mm, and FOV = 224 × 177 × 168 mm). Scan time for the anatomical scan was 296 s. A radiologist evaluated all T1 scans and no anomalous findings were reported. With respect to image processing, cortical reconstruction was performed with the longitudinal stream (Reuter et al., 2012) in FreeSurfer 6.0.0, a program for cortical surface reconstruction and volumetric segmentation (http://surfer.nmr.mgh.harvard.edu/). The procedure and technical details are described elsewhere (Fischl et al., 1999a, b; Reuter et al., 2012). To extract reliable volume estimates, an unbiased within-subject template space and image (Reuter and Fischl, 2011) is created using robust inverse consistent registration (Reuter et al., 2010). Several processing steps, such as skull stripping, Talairach transformation, atlas registration as well as spherical surface maps and parcellations are then initialized with common information from the within-subject template, significantly increasing reliability and statistical power (Reuter et al., 2012).

Parcellation of the cortex into gyral regions was based on the Desikan-Killiany-Tourville atlas (Klein and Tourville, 2012). This labelling process involved surface inflation (Fischl et al., 1999a) and registration to a spherical atlas based on subject specific cortical folding patterns (Fischl et al., 2004a,b).

Post-processing of the scan quality was conducted using a semi-automatic quality assessment tool, Qoala-T (Klapwijk et al., 2019). The Qoala-T tool rates a FreeSurfer processed scan on a scale of 0–100, where 0 indicates poor quality and 100 good quality. The Qoala-T tool recommends excluding scans with a rating below 30, manually inspect scans with a rating between 30 and 70 and include scans with a high rating above 70. This rating is based on FreeSurfer segmented data, including subcortical volumes, cortical surface area and thickness, and number of surface holes. All data at T1 data was manually checked, using the procedure described in Klapwijk et al. (2019). Results of the Qoala-T prediction model advised a manual quality check for nine participants at T2 and for five participants at T3. In addition, to confirm the accuracy of the model prediction, we randomly selected a subset (20%) of the scores >70 at T2 and T3 for manual quality checks. In total, MRI data of five participants was excluded based on quality checks. Specifically, from the manually checked T1 data, two participants were excluded. MRI data of 2 participants at T2 and T3 was excluded based on the Qoala-T model and data of one participant was excluded at T3 from the random subset. All manual scan quality checks were conducted by E.K. and L.W. In total, 451 scans were of good quality; 112 participants had usable scans at three waves, 40 participants had scans at two waves, 35 participants had a scan at one wave, 11 participants had zero usable scans. Three individuals did not report on their age and could not be used in our longitudinal growth curve analyses. Therefore, we conducted all our longitudinal structural brain development analyses on 198 minus 14 participants (i.e., N = 184) due to no usable scans or missing age.

#### 2.3.1 Brain regions of interest

**Fig. 1:**
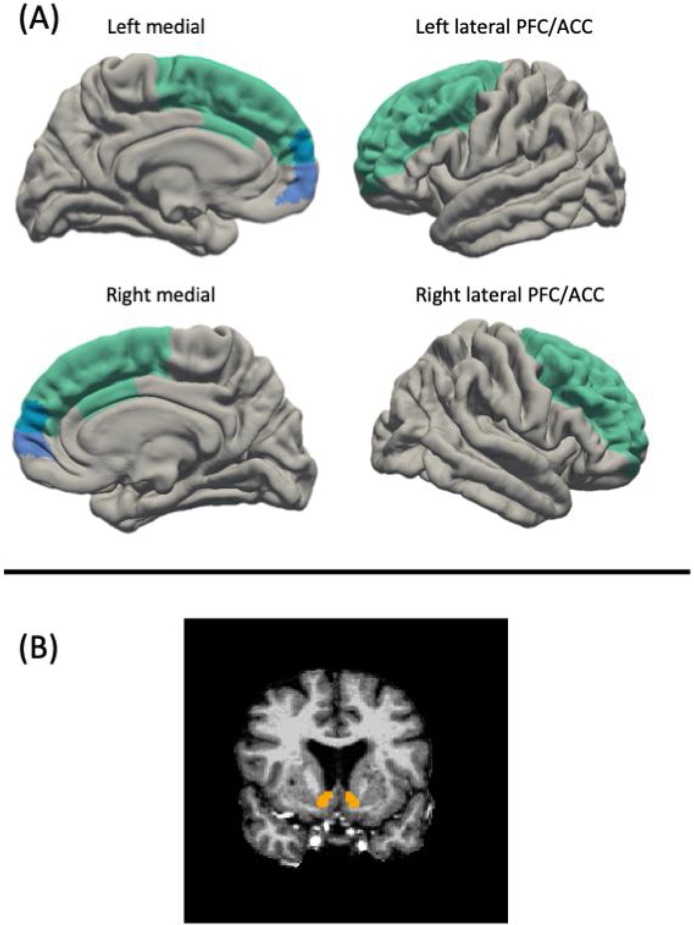
Regions of interest for the current study. Panel A) shows medial and lateral views of the lateral PFC/ACC (in green), which consisted of the combination of the rostral middle frontal gyrus, caudal middle frontal gyrus, caudal anterior cingulate gyrus, and superior frontal gyrus from the Desikan-Killiany-Tourville cortical parcellation atlas (Klein and Tourville, 2012) and the medial PFC (in blue), which was defined as Brodmann area 10 (mBA10) from Mills et al., 2014a,b; https://figshare.com/articles/Social_Brain_Freesurfer_ROIs/726133). Panel B) displays the nucleus accumbens, which consisted of ‘Accumbens area’ from the FreeSurfer Aseg atlas (Fischl et al., 2002). For interpretation of the references to colour in this figure legend, the reader is referred to the web version of this article.

The lateral PFC/ACC ROI was defined by combining parts of the PFC and ACC subdivisions similar to Becht et al. (2018) and Mills et al., 2014a). Specifically, this lateral PFC/ACC ROI was defined using the Desikan-Killiany-Tourville cortical parcellation atlas (Klein and Tourville, 2012) by combining the following subdivisions: rostral middle frontal gyrus, caudal middle frontal, caudal anterior cingulate, and superiorfrontal. For the medial prefrontal cortex ROI we used the Brodmann area 10 (mBA10) as a proxy of the mPFC, similar to Mills et al., 2014b; https://figshare.com/articles/Social_Brain_Freesurfer_ROIs/726133). We examined volume, surface area and cortical thickness for lateral PFC/ACC and medial PFC. NAcc was defined using the Freesurfer volumetric segmentation procedure. We computed bilateral measurements of surface area and volume by computing the mean of left and right hemispheres. To compute bilateral measurements of cortical thickness, we took the size of each region into account (for details see van der Meulen et al., 2020). See Fig. 1 for the three regions of interest (i.e., lateral PFC/ACC, medial PFC and nucleus accumbens.

### 2.4 Missing value analyses

Missing value analyses indicated that 70% of all possible datapoints were completed by the participants across three waves. Little’s missing completely at random (MCAR) test showed a chi-square (χ^2^/df) of 0.97, demonstrating that it is unlikely that findings were biased as a result of missing values. Subsequent attrition analyses revealed that adolescents who participated at T1 but dropped out over the course of the study at T3 did not differ on any of the background variables and study variables at T1 (i.e., age, identity variables and structural brain, *F*(11, 114) = 1.05, *p* = .408, η^2^ = 0.09. Besides, drop out was not related to participants’ sex, χ^2^ (1, *N =* 160) = 1.11, *p* = .293. Hence, we included all participants with and without missing values in our analyses and handled missing data in *Mplus* 8.2 (Muthén and Muthén, 2010), using full information maximum likelihood (FIML).

### 2.5 Statistical analyses

For our first aim, we examined the number of identity subgroups and the shape of their developmental trajectories of identity commitment levels, commitment fluctuations, and reconsideration. To this end, we conducted a multivariate latent class growth curve analyses (LCGA; Jung and Wickrama, 2008) on three waves in *Mplus* (Muthén and Muthén, 2010). We applied the TSCORES option in *Mplus* to account for the relatively large age range at each wave that is inherent to an accelerated longitudinal design as was used in the present study (Mehta and West, 2000). See online supplement S1 for details on the TSCORES option. To determine the number of latent classes that best fitted the data, we used the 1) Bayesian Information Criterion (BIC; Schwarz, 1978, lower BIC value indicates a better fitting model), (2) the Akaike Information Criterion (AIC; Akaike, 1974), (3) entropy, a measure of qualification certainty, should be acceptable (i.e., .75 or higher; Reinecke, 2006), (4) every class needs to cover at least 20% of the sample for meaningful interpretation and subsequent analyses. Finally, we will (5) consider the interpretability of the content of the classes. For example, if an additional class increases model fit, but is found to be a slight variation of a class solution with 1 class less, we will choose the most parsimonious class solution.

For our second aim, we examined the shape of structural brain developmental trajectories, by conducting a series of latent growth curve models (LGMs; Duncan and Duncan, 2009). We conducted seven separate LGMs for each brain region of interest and (for lateral PFC/ACC and medial PFC) for volume, surface area and cortical thickness, separately. Thus, for each ROI we used the volume, surface area, and cortical thickness observed scores per time point as input for the latent growth models. We compared model fit of the linear versus quadratic growth models with the BIC and AIC. Similar to the LCGA analyses, we applied the TSCORES option in the LGMs in order to account for the age heterogeneity at each wave. In all LGMs we controlled the intercepts and slopes of brain development for sex differences. If boys and girls did not differ on structural brain intercept and slopes, we dropped drop sex as a covariate from the final growth models.

For our third aim, we tested differential development brain trajectories across different identity classes. To this end we will use the continuous identity class probabilities that were obtained from the LCGA analyses (aim 1). Next, we added these correlations between the identity class probabilities and the intercept and linear slopes of structural brain development to the LGMs as specified for aim 2. We estimated all our models with the robust MLR estimator to account for non-normal distributions of data (Muthén and Muthén, 2010), including the identity reconsideration scores and the identity class probabilities.

Given the substantial number of tested models, we applied the false discovery rate (FDR) correction as described by Benjamini and Hochberg (1995). We report these corrected p-values in our results section. Please find all original p-values and the FDR corrected p-values as online supplementary material S4.

## 3 Confirmatory results

### 3.1 Heterogeneity in identity developmental trajectories

Our first aim was to examine whether there are subgroups of individuals with distinct developmental trajectories of commitment and reconsideration level and fluctuations across three waves. The LCGA models with the TSCORES option did not converge. Alternatively, following our pre-registered analytical strategy, we controlled the intercept and slopes for age. Importantly, however, we deviated from our pre-registered analytical procedure in two ways. First, we were able to use the bootstrapped likelihood ratio test (BLRT; McLachlan and Peel, 2004) as an additional fit measure to decide on the number of classes. That is because the BLRT is available when conducting a regular LCGA but not with the TSCORES option. Second, we added the Akaike Information Criterion (AIC; Akaike, 1974) and sample size adjusted BIC (ssaBIC; Sclove, 1987) in addition to the BIC, because we observed inconstancies between the BIC and AIC values when testing the number of identity classes. Given that model selection with mixture models involves a certain level of uncertainty, we followed recommendations to report all available fit information, especially when fit indexes are not unanimous (Grimm et al., 2017). Moreover, the ssaBIC has been found to outperform other fit indices such as the AIC and entropy (Tein et al., 2013). We therefore added these fit measures to evaluate the number of classes.

Consistent with our pre-registered hypothesis, latent class growth curve analyses revealed that a 2-class solution provided the best fit to the data. Although the BIC of the 1-class solution (2398.53) was lower than the BIC of the 2-class solution (2464.79), results of the other model fit comparison indices supported the 2-class solution. That is, the ssaBIC (2347.70) and AIC (2354.39) of the 2-class solution were lower compared to the ssaBIC (2393.65) and AIC (2479.09) of the 1-class solution. In addition, the bootstrapped likelihood ratio test indicated that a 2-class solution provided a significantly better fit than the 1-class solution (*p* = <.001). Finally, entropy (.884) of the 2-class solution was also high. Compared to the 3-class solution, the 2-class solution provided the best fit as well. While the AIC and ssaBIC of the 3-class solution were lower (2347.20 and 2338.70 respectively), the BIC of the 2-class solution was lower (2464.79), entropy was lower (.72 vs .88), and the bLRT showed a nonsignificant improvement for a 3-class solution, compared to the 2-class solution (*p* = .062). Taken together, based on the model comparison indices, a 2-class solution provided the best fit with the data.

The first identity synthesis class (82%), is characterized by 1) relatively high and stable commitment levels, 2) stable commitment fluctuations, and 3) stable low reconsideration across waves. The second identity moratorium class (18%) is characterized by 1) relatively high and stable commitment levels, 2) stable commitment fluctuations, and 3) relatively high reconsideration. Please note that one of the pre-registered criteria to determine the number of latent classes was that every class needed to cover at least 20% of the sample. One of our identified latent classes was below this pre-specified minimum because it included 18% of the sample. However, we decided to continue with our subsequent analyses, because the other fit indices supported the 2-class solution. As an additional (non-preregistered exploratory) validity check, we examined whether individuals in the identity moratorium class differed on the levels of internalizing and externalizing problem behaviors. Pleases find these results in online supplement S2.

**Fig. 2:**
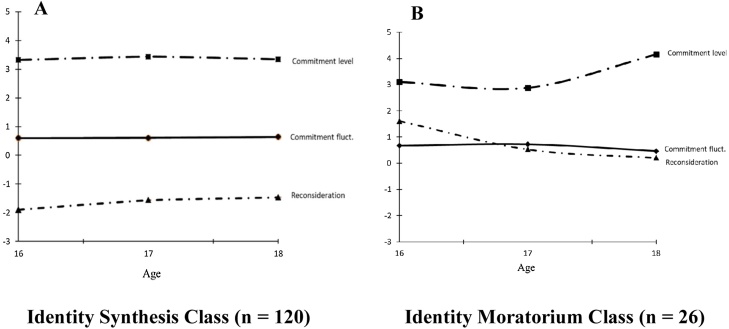
Final two class solution of the latent class growth curve analysis (LCGA) showing the estimated trajectories fo commitment level, commitment fluctuations, and reconsideration. For reasons of clarity we visualized the estimated trajectories for all three variables and not the raw individual level trajectories. Average age for each wave is presented on the x-axis.

The LCGA model with the TSCORES option did not converge. Alternatively, following our pre-registered analytical strategy, we controlled the intercept and slopes for age. In the final 2-class model, we only included the significant effect of age on the intercept of reconsideration, which indicated that older adolescents reported a higher reconsideration intercept, *b* = 0.08, *p* = .006. This model provided a better fit compared to the model including all other non-significant age effects. The estimated intercept and slope growth parameters of the final 2-class solution can be found in Table 1. Growth trajectories of commitment level, commitment fluctuations and reconsideration in both subgroups are presented in Fig. 2.

**Table 1:** Parameter Estimates of Intercept and Slope Factors of Latent Classes for Identity Subgroups.

		Identity Synthesis Class (82 %)			Identity Moratorium Class (18 %)	
		*M*	*SE*		*M*	*SE*
*Commitment level*						
Mean intercept		3.32*** _a_	0.08		3.11*** _a_	0.14
Mean linear slope		0.22 _a_	0.20	–	−0.49 _a_	0.42
Mean quadratic slope	–	−0.10 _a_	0.10		0.26 _a_	0.20
*Commitment fluctuations*						
Mean intercept		0.60*** _a_	0.05		0.67*** _a_	0.08
Mean linear slope	–	0.00 _a_	0.15		0.22 _a_	0.32
Mean quadratic slope		0.01 _a_	0.07	–	0.16 _a_	0.17
*Reconsideration*						
Mean intercept	–	1.89***	0.46		1.61*	0.62
Mean linear slope		0.44_a_	0.41	–	1.46_a_	1.45
Mean quadratic slope	–	0.12 _a_	0.23		0.38 _a_	0.84

*Note*. *p <.05, **p <.01, ***p <.001. Means with the same subscript do not differ significantly from one another. Thus, means without a subscript also differ significantly from one another. Note that the subscripts apply to each growth function in each identity domain separately (e.g., differences between mean intercepts of commitment level of the two identity classes). All *p*’s <.05.

Concerning the level and developmental change in commitment and reconsideration processes, results revealed no significant linear or quadratic effects for commitment level, commitment fluctuations and reconsideration in both identity classes. Moreover, both identity classes did not significantly differ in the change parameters (i.e., linear or quadratic slopes) on the different identity dimensions. However, as can be seen Table 1, the intercepts of reconsideration differed significantly between the identity classes such that individuals in the identity moratorium class showed a higher reconsideration intercept relative to individuals in the identity synthesis class.

### 3.2 Structural brain development of lateral PFC/ACC and medial PFC and nucleus accumbens

**Fig. 3:**
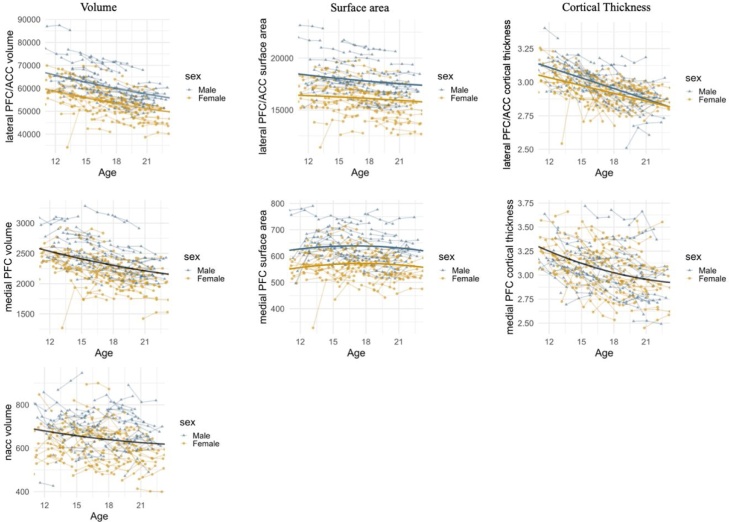
Observed individual trajectories for each region of interest. PFC = prefrontal cortex; ACC = anterior cingulate cortex; NAcc = nucleus accumbens.

Next, we conducted a series of LGMs with TSCORES to examine the developmental shape of the lateral PFC/ACC, the medial PFC and NAcc from early adolescence into young adulthood (ages 10–25 years). Fit indices of the best fitting models (linear vs quadratic) across three waves are presented in online supplementary material Table S1. Growth parameter estimates of the final growth models and raw and estimated developmental trajectories are presented in online supplementary Table S2 and Fig. 3, respectively.

#### 3.2.1 Lateral PFC/ACC volume, surface area, cortical thickness

A quadratic growth model provided the best fit to the lateral PFC/ACC volume and surface area data, whereas cortical thickness of the lateral PFC/ACC was best described by a linear model. Regarding the developmental shape, the lateral PFC/ACC volume showed a linear decrease from early to late adolescence and levelled off into young adulthood (indicated by a positive quadratic effect). Lateral PFC/ACC surface area was stable from early adolescence into young adulthood. Cortical thickness of the lateral PFC/ACC showed a linear decrease across adolescence into young adulthood.

#### 3.2.2 Medial PFC volume, surface area, cortical thickness

Medial PFC volume, surface area and cortical thickness were all best described by a quadratic growth model. Regarding the developmental shape, results indicated a linear decrease of medial PFC volume over time. Medial PFC surface area was stable from early adolescence into young adulthood. Medial PFC cortical thickness decreased linearly across adolescence and levelled off into young adulthood.

#### 3.2.3 Nucleus accumbens

The development of the NAcc was best described by a quadratic model. Yet, concerning the specific developmental shape, the mean of the linear and quadratic slope was not significantly different from zero. Thus, results revealed stable volume levels across development.

#### 3.2.4 Sex differences in brain development

To examine whether boys and girls differed on structural brain intercepts and change, we added sex as predictors of the intercept and slopes. Girls showed a lower intercept of lateral PFC/ACC volume compared to boys, *b* = -0.81, *p* = .011. Similarly, girls showed a lower intercept of lateral PFC/ACC surface area compared to boys, *b* = -3.43, *p* = .048, and a lower intercept of lateral PFC/ACC cortical thickness compared to boys, *b* = -0.158, *p* = .048. Finally, girls had a lower intercept of the medial PFC surface area compared to boys, *b* = -0.794, *p* = .025. Girls and boys did not differ on intercept and rate of change (i.e., slope) of the other structural brain region of interests.

### 3.3 Structural brain development of adolescents in different identity trajectory subgroups

Our third and final aim was to test whether the two identified identity classes differed in the structural brain development of lateral PFC/ACC, the medial PFC and NAcc gray matter. To this end, we correlated the class probability of belonging to the identity moratorium class with the intercept and slopes of structural brain development. These correlations can be found in Table S3. In the current early exploratory stages of research on the linkages between structural lateral PFC/ACC and medial PFC and identity formation we are unable to differentiate our expectations between volume, surface area and cortical thickness separately. Therefore, we had similar expectations for the linkages between lateral PFC/ACC and medial PFC volume, surface area, cortical thickness and identity.

#### 3.3.1 Lateral PFC/ACC and medial PFC volume, surface area, cortical thickness

We predicted that individuals who were more likely to belong to the identity moratorium subgroup to show lower intercepts of the lateral PFC/ACC and medial PFC volume, surface area, and cortical thickness. Contrary to these predictions, results revealed no intercept differences of lateral PFC/ACC and medial PFC volume, surface area, and cortical thickness. Regarding linear structural brain changes, we expected that individuals who were more likely to belong to the moratorium class would show a faster linear decline of lateral PFC/ACC and medial PFC volume, surface area, and cortical thickness. Contrary to our expectations, we found no support for the link between belonging to the moratorium class and the development of lateral PFC/ACC and medial PFC volume, surface area, and cortical thickness.

#### 3.3.2 Nucleus accumbens

Consistent with our predictions, those adolescents who were more likely to belong to the moratorium class showed a lower NAcc intercept, *b* = -0.12, *p* = .029. Concerning the development of NAcc, we predicted that individuals with a higher likelihood of belonging to the moratorium class showed faster decreasing NAcc volume levels across adolescence into young adulthood. Contrary to our predictions, results revealed that a higher likelihood of belonging to the moratorium class correlated with a less steep decline in NAcc volume over time, *b* = 0.06, *p<*.001. As an additional sensitivity check, we reconducted the analyses by applying listwise deletion to examine whether dropping those individuals with missing scans affected the results. This was not the case as the results were virtually the same. Therefore, we report the results that include all participants with and without missing data.

## 4 Exploratory results

The absence of relations between identity subgroups and trajectories of medial and lateral PFC/ACC development was unexpected. Therefore, in addition to our pre-registered confirmatory analyses, we conducted non-pre-registered exploratory analyses on the longitudinal associations between in-depth exploration and lateral PFC/ACC and medial PFC. That is because in-depth exploration was previously predicted by the lateral PFC/ACC (Becht et al., 2018), but was not part of our pre-registered analyses. Therefore, we conducted six multivariate exploratory latent growth curve analyses on the longitudinal associations between lateral PFC/ACC and medial PFC volume, surface area, and cortical thickness and in-depth exploration to test whether we could replicate previous findings by Becht et al. (2018). Specifically, we tested whether the intercept and linear slopes of the lateral PFC/ACC and medial PFC correlated with the intercept and linear slope of in-depth exploration, respectively. In addition, we examined whether the intercept of lateral PFC/ACC and medial PFC predicted change of in-depth exploration and vice versa, whether the intercept of in-depth exploration predicted change of the lateral PFC/ACC and medial PFC.

The univariate growth model of in-depth exploration revealed stable levels of in-depth exploration over time, with a mean intercept of *b* = 3.14, *p* < .001, and mean slope *b* = 0.30, *p* = .061. For reasons of model complexity of the multivariate growth models, we did not examine non-linear development of in-depth exploration. Table S4 shows the associations between the intercepts and slopes of the lateral PFC/ACC and medial PFC and the intercept and linear slope of in-depth exploration. We tested whether adolescents with higher intercepts and a less steep decline in lateral PFC/ACC and medial PFC would reveal higher baseline levels (i.e., intercepts) and relative stronger increases of in-depth exploration over time.

*Lateral PFC/ACC volume.* Multivariate growth curve models revealed that the intercept of lateral PFC/ACC volume did not relate to the linear slope of in-depth exploration. The intercept-intercept, and slope-slope associations were not significant either. However, a higher intercept of in-depth exploration predicted a less steep decline in lateral PFC/ACC volume over time.

*Lateral PFC/ACC surface area.* A higher intercept of lateral PFC/ACC surface area correlated with a lower intercept of in-depth exploration. In addition, we found evidence of correlated growth such that a less steep decline of the lateral PFC/ACC surface area correlated with a less positive slope of in-depth exploration. Moreover, a relatively higher intercept of the lateral PFC/ACC surface area predicted a linear increase of in-depth exploration. Finally, a higher intercept of in-depth exploration predicted a steeper decline in lateral PFC/ACC surface area.

*Lateral PFC/ACC cortical thickness.* Those adolescents with a relatively a higher lateral PFC/ACC thickness intercept predicted a less steep increase of in-depth exploration over time. We found no evidence for correlated growth (slope-slope associations).

*Medial PFC volume*. Adolescents with a higher medial PFC intercept showed a steeper increase of in-depth exploration over time. In addition, a higher intercept of in-depth exploration predicted a relatively less steep decline of medial PFC volume.

*Medial PFC surface area.* None of the associations between the intercept and slopes of medial PFC surface area were significant.

*Medial PFC cortical thickness.* Adolescents with a relatively higher intercept of medial PFC cortical thickness showed a relatively faster increase of in-depth exploration. None of the other associations (i.e., intercept-intercept, slope-slope and intercept-slope associations) were significant.

In sum, five out of eight significant associations were consistent with our overall hypothesis that those adolescents with higher lateral PFC/ACC and medial PFC levels (indicated by a higher intercept and/or less steep decline over time) showed a relatively higher intercept and/or steeper increase of in-depth exploration. Contrary to our expectations, a higher intercept of the lateral PFC/ACC surface area was related to a lower intercept of in-depth exploration, and not with a higher intercept of in-depth exploration. Besides, a less steep decline of the lateral PFC/ACC surface area correlated with less increase of in-depth exploration, rather than the expected stronger increase. Finally, a higher intercept of the lateral PFC/ACC cortical thickness predicted less increase of in-depth exploration over time, rather than the expected stronger increase of in-depth exploration over time.

## 5 Discussion

The goal of this longitudinal study was to examine whether structural brain developmental patterns differ between adolescents who experience daily identity uncertainty compared to adolescents who develop stable and strong identity commitments. Behaviorally, we confirmed two subgroups of adolescents, those with stable high commitments, labeled the *identity synthesis class*, and those adolescents in a subgroup labeled the *identity moratorium class*, with relatively higher levels of daily identity reconsideration. Longitudinal structural brain developmental patterns confirmed most of our hypotheses of decreasing lateral PFC/ACC and medial PFC volume, and cortical thickness and stable NAcc volume. Lateral PFC/ACC and medial PFC surface area were stable across ages 10–25 years. When testing the longitudinal associations between these structural brain developmental trajectories and identity subgroups, we observed lower baseline levels and less decline in NAcc volume for adolescents in the identity moratorium class compared to adolescents in the identity synthesis class. Lateral PFC/ACC and medial PFC developmental trajectories of volume, surface area, and cortical thickness did not differ across identity subgroups. However, additional exploratory analyses revealed that adolescents with higher levels of lateral PFC/ACC and medial PFC volume, surface area and cortical thickness and delayed maturation (i.e., less steep declines) showed higher levels and greater increases of in-depth exploration of identity commitments.

### 5.1 Daily heterogeneity in identity development

An important question concerns the individual differences in identity developmental patterns in relation to developmental outcomes (Klimstra and Denissen, 2017). Prior longitudinal studies that used annual identity assessments revealed individual differences in identity formation processes (e.g., Luyckx et al., 2008; Meeus et al., 2012), but a remaining question is whether and how identity formation processes differ between adolescents and fluctuate across days. Consistent with one prior study (Becht, Nelemans et al., 2016), we identified two identity subgroups that differed in their daily certainty and uncertainty about their identity commitments. Specifically, adolescents in the largest (i.e., 82 %) *identity synthesis* class were characterized by strong identity commitments and low daily identity reconsideration. This daily identity profile was very similar to adolescents in an identity achievement or (for)closure status (i.e., relatively high commitment levels and low identity reconsideration), as observed in longitudinal studies based on annual data (Crocetti, 2017; Hatano and Sugimura, 2017; Meeus et al., 2012).

Individuals in the second smaller (i.e., 18% of the total sample) identity moratorium subgroup reported similarly high identity commitment levels compared to individuals in the identity synthesis class. Yet, they showed significantly higher ongoing daily reconsideration of these commitments as well. As such, this subgroup with relatively strong commitments and high reconsideration seems to resemble the previously found moratorium identity status (Kroger et al., 2010; Meeus, 2011), and extends prior work by showing the existence of an identity moratorium status at the daily level.

Contrary to our expectations we did not find significant changes in identity commitments and reconsideration over time as found in prior work (Becht, Nelemans et al., 2016). However, the observed slopes of commitment level showed the expected direction. That is, commitments for adolescents in the identity moratorium class decreased, and increased for adolescents in the identity synthesis class. Besides, commitment levels were lowest, and reconsideration was highest between 15–17 years of age for adolescents in the identity moratorium class. This dip of identity commitments and peak of identity reconsideration was observed in prior work as well (Becht, Nelemans et al., 2016). In addition, we expected that identity classes would differ significantly on commitment baseline levels, which was not confirmed in this study. Yet, the observed commitment intercept was lower for adolescents in identity moratorium, compared to the identity synthesis class. Possibly, a larger sample would have provided more power to detect significant differences in intercepts and slopes. Future studies are therefore needed to examine these identity trajectories in a larger longitudinal sample.

### 5.2 Structural brain development

At the neural level we examined structural brain development for adolescents with ongoing daily identity uncertainty versus adolescents with stable and strong identity commitments across days. First, on average across participants, we found non-linear decreases in structural developmental trajectories of lateral PFC/ACC and medial PFC and NAcc that were mostly consistent with previous longitudinal studies. That is, lateral PFC/ACC and medial PFC volume linearly decreased across adolescence with a slight upward trend for the medial PFC (Becht et al., 2018; Mills et al., 2014a,b; Tamnes et al., 2017). Cortical thickness of lateral PFC/ACC and medial PFC showed a steady predicted linear decline (Wierenga, Langen, Oranje et al., 2014), that levelled off in the medial PFC (Tamnes et al., 2017).

In the current data set, surface area of the lateral PFC/ACC and medial PFC was stable rather than showing the previously identified and hypothesized (non-) linear decline (e.g., Becht et al., 2018; Mills et al., 2014a,b). While it has been found that surface area shows the smallest decline across development compared to volume and cortical thickness, most studies report decreasing surface area levels (Tamnes et al., 2017). Possibly, different age ranges between studies explain part of the inconsistencies. For instance, Tamnes et al. (2017) reported a flatter slope in a longitudinal study with a larger age range (i.e., 7–30 years) that included emerging adults, compared to longitudinal studies with a narrower age range (i.e., 8–27 years) that reported faster declines. Future studies are needed to further examine the development of lateral PFC/ACC and medial PFC surface area across a larger age range to study ongoing development from childhood into adulthood.

As predicted, NAcc volume was stable across development in our sample with a similar age range (10–25 years) as some previous studies (12–22 years; Becht et al., 2018; 8–22 years; Herting et al., 2018). Yet, possibly depending on the exact age range that is studied, evidence on the developmental shape of NAcc remains mixed, with studies reporting decreases across ages 7–30 years (Mills et al., 2014a), and across ages 8–26 years (Urosevic et al., 2012; Wierenga et al., 2018; Wierenga, Langen, Ambrosino, et al., 2014). Future studies that include a larger age range from childhood well into adulthood are needed to further examine the developmental patterns of subcortical brain development.

### 5.3 Structural brain development and heterogeneity in daily identity development

A key question is whether subgroups of adolescents with heterogenous identity formation patterns show differential brain development (Becht and Mills, 2020; Crone and Fuligni, 2020). Studies on structural brain development have reported considerable variability between individuals (Herting et al., 2018; Mills et al., 2016; Tamnes et al., 2017), but how this relates to developmental outcomes is not well understood (Becht and Mills, 2020; Rosenberg et al., 2018).

As predicted, adolescents in identity moratorium showed lower NAcc volume baseline levels (Becht et al., 2018). Prior work also revealed that higher NAcc volume levels relate to increased goal-setting and motivated behavior (Nemmi et al., 2016; Urosevic et al., 2012), and was therefore expected to facilitate the process of forming strong and self-defined identity commitments. Contrary to our hypothesis, adolescents in identity moratorium showed a more positive linear slope, indicating delayed maturation and thus relatively higher NAcc volume levels across waves. Please note that, on average, the linear slope of the NAcc was negative but not significant. As a result, the positive correlation between identity moratorium and the NAcc slope indicates that individuals in identity moratorium have a less negative NAcc linear slope (i.e., delayed maturation rather than the predicted faster decline) compared to individuals in the identity synthesis class. Thus, while previous findings tentatively suggested that delayed maturation of NAcc volume would be associated with a stronger identity (Becht et al., 2018), we found the opposite pattern. At the same time, however, this unexpected finding is consistent with a study that found relatively slower volume decreases of subcortical structures (i.e., amygdala) in adolescents from disadvantaged neighbourhoods, which are characterized by high stress levels (Whittle et al., 2017). Thus, stressful environments have been related to atypical slower rates of subcortical development. Similarly, adolescents in identity moratorium reported ongoing daily identity uncertainty, which is very stressful as well, as evidenced by increased anxiety levels (Becht, Nelemans et al., 2016). Consistent with slower rates of amygdala development for individuals living in stressful environments, adolescents with high levels of daily identity stress showed slower rates of NAcc volume development as well. Together, these findings confirm the involvement of NAcc volume levels and specific developmental patterns of NAcc for adolescents with different levels of daily identity certainty and uncertainty. Yet, future studies are needed to replicate our findings.

Contrary to our hypotheses, developmental trajectories of the lateral PFC/ACC and medial PFC did not differ between identity formation subgroups. Although our initial pre-registered hypotheses predicted longitudinal linkages between processes of commitment and reconsideration and lateral PFC/ACC and medial PFC, these unexpected null results led us to conduct additional exploratory analyses to examine the longitudinal linkages between lateral PFC/ACC and medial PFC and in-depth identity exploration levels and change. Adolescents’ in-depth identity exploration is considered the most reflective and cognitive process during identity development because it taps into adolescents’ reflection on their identity commitments (Crocetti et al., 2008; Meeus et al., 2010). As such, this identity process may be related to lateral PFC/ACC and medial PFC trajectories which are considered key regions to support cognitive processes that are associated with identity development (Pfeifer and Peake, 2012). Our results revealed indeed that those adolescents with relatively higher baseline levels and delayed maturation (i.e., less steep declines) of lateral PFC/ACC and medial PFC (volume, surface area and cortical thickness) reported higher levels and greater increases of in-depth exploration. These findings of delayed maturation of the lateral PFC/ACC and medial PFC predicting increasing in-depth exploration are consistent with the assumption that increased neural plasticity provides adolescents with opportunities to learn and explore their identity (Blakemore & Mills, 2014). Besides, our results are consistent with functional imaging studies in adults and adolescents that showed increased activity in medial PFC when participants think about themselves (Summerfield et al., 2009; van der Cruijsen et al., 2018; Van der Cruijsen et al., 2019).

To our knowledge no prior studies tested the longitudinal linkages between structural medial PFC and identity development, but the medial PFC has been linked to other behavioral outcomes. Some studies indicated that faster decreasing medial PFC volume related to less rebellious behavior (Blankenstein et al., 2019), and a more mature personality profile, indicated by higher emotional stability, and conscientiousness (Ferschmann et al., 2018). Yet, other studies indicated that delayed maturation (slower cortical thinning) predicted prosocial behaviors. Specifically, adolescents with higher prosocial behaviors showed initially faster cortical thinning of the medial PFC, which slowed down to less thinning during the transition to adulthood (Ferschmann et al., 2019). Similarly, delayed maturation in lateral PFC/ACC predicted more in-depth exploration in previous work (Becht et al., 2018), and the present study. These inconsistencies between studies call for future studies that further examine differential developmental patterns of medial PFC in relation to variability in behavioral outcomes.

### 5.4 Strengths and limitations

We acknowledge the strengths and limitations of this study. First, we collected intensive longitudinal daily diary data to tap into the level and day-to-day identity fluctuations. The longitudinal design allowed us to examine individual differences in starting levels and changes in structural brain regions and relate these individual differences in brain development to fluctuations in behavioral development (Becht and Mills, 2020; Foulkes and Blakemore, 2018; Rosenberg et al., 2018).

Inevitably, the current study also had some limitations. First, we were limited in our assessment of identity in the educational domain and did not assess identity formation processes in other identity domains. Although the patterns of daily identity formation were similar in the educational and interpersonal identity domain (Becht, Nelemans et al., 2016), future studies are needed to test for differential brain development in interpersonal identity domain subgroups. Second, the number of participants in the identity moratorium class was relatively low, which may have masked additional differences in brain development. Future studies that include a larger sample are needed to replicate our findings. Third, a remaining key question for future studies concerns the temporal order of brain and identity processes (Hamaker et al., 2015). For instance, does a within-person increase in medial PFC volume precede within-person increases of in-depth exploration over time, or vice versa? Fourth, identity development does not take place in a social void, but in close interaction with important others. Prior work revealed, for instance, that the development of strong identity commitments and a stable self-concept developed in the context of high-quality relationships with parents and peers (e.g., Becht, Nelemans, Branje et al., 2017; Becht, Nelemans, Dijk et al., 2017; Crocetti et al., 2017). Therefore, future studies are needed that examine the longitudinal interplay between quality of relationships, brain development and identity development across adolescence, including multiple indices beyond single-item identity scales and across multiple identity domains (e.g., interpersonal identity) to capture these complex developmental processes. Fifth, the majority of participants in this sample followed relatively higher secondary and tertiary educational levels compared to the Dutch national average. As a result, findings may not generalize to individuals from lower educational levels and families with a lower socioeconomic status. Future studies are needed to examine longitudinal associations between structural brain development and identity development in a more diverse sample to test for generalizability of findings.

In conclusion, this longitudinal study examined whether the subgroup of adolescents characterized by high daily identity uncertainty in the educational identity domain shows differential structural brain developmental trajectories of lateral PFC/ACC and medial PFC and NAcc. Findings confirm the contribution of individual differences in NAcc development in the process of educational identity formation, with lower baseline levels and slower declines in NAcc development for adolescents with high identity uncertainty. Moreover, delayed maturation in lateral PFC/ACC and medial PFC developmental trajectories predicted increasing in-depth exploration, supporting the contribution of these brain structures in the process of adolescents’ active exploration and reflection upon their identity commitments. The current findings highlight the importance to study how variability in brain developmental trajectories are associated with variability in identity formation processes and provide new insights in the possible neurobiological architecture of adolescents’ identity formation.
