# Corrigendum to “Longitudinal associations between structural prefrontal cortex and nucleus accumbens development and daily identity formation processes across adolescence” [Dev. Cogn. Neurosci. 46 (December) (2020) 100880]

**DOI:** 10.1016/j.dcn.2020.100890

**Published:** 2021-01-06

**Authors:** Andrik I. Becht, Eduard T. Klapwijk, Lara M. Wierenga, Renske van der Cruijsen, Jochem Spaans, Laura van der Aar, Sabine Peters, Susan Branje, Wim Meeus, Eveline A. Crone

**Affiliations:** a Erasmus School of Social and Behavioural Sciences, Erasmus University Rotterdam, the Netherlands; b Research Center Adolescent Development, Utrecht University, the Netherlands; c Brain and Development Research Center, Leiden University, the Netherlands

The authors regret that Table 1 contained some minor errors and should have appeared as follows:

**Table 1:** Parameter Estimates of Intercept and Slope Factors of Latent Classes for Identity.

	Identity Synthesis Class (82 %)		Identity Moratorium Class (18 %)	
	*M*	*SE*	*M*	*SE*
*Commitment level*				
Mean intercept	3.32*** _a_	0.08	3.11*** _a_	0.14
Mean linear slope	0.22 _a_	0.20	−0.49 _a_	0.42
Mean quadratic slope	−0.10 _a_	0.10	0.26 _a_	0.20
*Commitment fluctuations*				
Mean intercept	0.60*** _a_	0.05	0.67*** _a_	0.08
Mean linear slope	−0.00 _a_	0.15	0.22 _a_	0.32
Mean quadratic slope	0.01 _a_	0.07	−0.16 _a_	0.17
*Reconsideration*				
Mean intercept	−1.89***	0.46	1.61*	0.62
Mean linear slope	0.44_a_	0.41	−1.46_a_	1.45
Mean quadratic slope	−0.12 _a_	0.23	0.38 _a_	0.84

*Note*. *p <.05, **p <.01, ***p <.001. Means with the same subscript do not differ significantly from one another. Thus, means without a subscript also differ significantly from one another. Note that the subscripts apply to each growth function in each identity domain separately (e.g., differences between mean intercepts of commitment level of the two identity classes). All *p*’s <.05.

The authors would like to apologise for any inconvenience caused.

