# Supplementary material for: Longitudinal associations between structural prefrontal cortex and nucleus accumbens development and daily identity formation processes across adolescence
Source: Dev Cogn Neurosci. 2020 Nov 11;46:100880. doi: 10.1016/j.dcn.2020.100880 (PMC7677671; doi:10.1016/j.dcn.2020.100880)

**Supplementary material**

Online supplementary material S1

Because adolescents in both studies varied significantly in age at each measurement wave, we applied a model where individually varying times of observations could be estimated (i.e., TSCORES option in M*plus*, Muthén & Muthén, 1998-2012). In conventional LGM, it is assumed that data are collected at an identical set of fixed ages for all individuals (Mehta & West, 2000). In these models, slope factor loadings of a linear slope are fixed across all individuals to be 0, 1, 2, for Waves 1-3, respectively. However, with the TSCORES option the LGM takes into account heterogeneity in age at each measurement wave. That is, rather than defining linear growth with fixed factor loadings, age is now included in the model as a defining variable to scale the factor loadings and estimate the growth curve. Consider the example where we put the intercept at age 12 years. In this example, the starting point of the developmental trajectory is set at age 12 by fixing the factor loading for each observed measure for each individual to reflect deviation in years from age 12. Therefore, because adolescents vary in age at each measurement wave, the factor loadings are unique to each individual. For instance, an adolescent with an actual age of 12 years at the first measurement wave will obtain the linear slope factor loadings of 0, 1, and 2 for age 12, 13, and 14 years, respectively. However, an adolescent aged 15 years at the first measurement wave will obtain linear slope factor loadings of 3, 4, and, 5 for age 15, 16 and 17 years, respectively. Thus, each individual contributes to the estimation of parts of the growth trajectory for ages at which he or she does provide data (please see Mehta & West, 2000 for a detailed discussion of modelling individually varying times of observations).

Online supplementary material S2

As an additional (non-preregistered exploratory) validity check, we examined whether individuals in the identity moratorium class differed on the levels of internalizing and externalizing problem behaviors. We used the internalizing and externalizing subscales from the Strenghts and Difficulties Questionnaire (SDQ) as an index of psychosocial adjustment. We conducted a multivariate analyses of variance (MANOVA) with internalizing and externalizing problem behaviors at T1-T3 as dependent variables and class membership as independent variable. Results revealed a consistent pattern of higher internalizing and externalizing problem behaviors in each of the three waves for individuals in the identity moratorium class compared to individuals in the synthesis class, *F* (6, 125) = 3.32, *p* =.005, *η^2^* = .14. These patterns were very similar to previously reported adjustment differences of daily identity subgroups (Becht et al., 2016), and further substantiate the validity of the two identity classes and associated stress of ongoing daily identity uncertainty (Meeus, 2011; Van Doeselaar et al., 2018).

| Table S1  *Fit indices of the latent growth models for structural brain development of lateral PFC/ACC, medial PFC and nucleus accumbens* | | | | | |
| --- | --- | --- | --- | --- | --- |
| **Brain region** Linear model | | |  | Quadratic model | |
|  | AIC | ssaBIC |  | AIC | ssaBIC |
| Lateral PFC/ACC volume | 255.21 | 255.50 |  | **230.08** | **230.55** |
| Lateral PFC/ACC surface area | 835.17 | 835.46 |  | **817.33** | **817.80** |
| Lateral PFC/ACC thickness | **-950.55** | **-950.27** |  | -948.47 | -948.14 |
| Medial PFC volume | -429.95 | -429.66 |  | **-442.93** | **-442.45** |
| Medial PFC surface area | 481.74 | 482.03 |  | **473.27** | **473.75** |
| Medial PFC thickness | -561.59 | -561.30 |  | **-572.16** | **-571.68** |
| Nucleus accumbens | 705.57 | 705.85 |  | **704.37** | **704.85** |

*Note.* Preferred final models are depicted in bold. AIC=Aikaike information criterion;

ssaBIC= sample size adjusted Bayesian Information Criterion; PFC=prefrontal cortex.

PFC=prefrontal cortex; ACC=anterior cingulate cortex.

Table S2

*Unconditional Growth Parameters Estimates for lateral PFC/ACC,*

*medial PFC and nucleus accumbens*

| Mean Int. (*SE*). Mean LS (*SE*). Mean QS (*SE*) | | | | | |
| --- | --- | --- | --- | --- | --- |
| lPFC/ACC Vol. | 8.57 (0.43)*** | - | 2.38 (0.48)*** |  | 0.42 (0.13)* |
| lPFC/ACC SA. | 18.10 (0.74)*** | - | 0.70 (0.78) |  | 0.01 (0.22) |
| lPFC/ACC Thick. | 3.34 (0.03)*** | - | 0.22 (0.02)*** |  | - |
| mPFC Vol. | 3.21 (0.21)*** | - | 0.68 (0.24)* |  | 0.10 (0.07) |
| mPFC SA | 5.36 (0.63)*** |  | 0.87 (0.73) | - | 0.28 (0.21) |
| mPFC Thick. | 4.04 (0.19)*** | - | 0.85 (0.21)*** |  | 0.16 (0.06)* |
| Nucleus acc. | 8.13 (0.75)*** | - | 1.40 (0.85) |  | 0.24 (0.23) |
| *Note*. Int= intercept; LS= Linear slope; QS= Quadratic slope;  lPFC/ACC= lateral prefrontal cortex/anterior cingulate cortex. mPFC= medial prefrontal cortex; Vol. = volume; SA = surface area; Thick. = thickness;  **p<.*05. ***p*<.01. ****p*<.001 | | | | | |

| Table S3 *Covariances between the identity moratorium class probability and growth factors for structural brain development* | | | |
| --- | --- | --- | --- |
|  | Class probability of identity  moratorium class | | |
| *Lateral PFC/ACC vol.*  Intercept  Linear slope | | - | 0.01  0.01 |
| *Lateral PFC/ACC sa.* Intercept Linear slope | | - | 0.03  0.03 |
| *Lateral PFC/ACC thick.*  Intercept  Linear slope | | - | 0.01  0.01 |
| *Medial PFC vol.*  Intercept  Linear slope | | - | 0.02  0.02 |
| *Medial PFC sa.*  Intercept  Linear slope | | - | 0.01  0.01 |
| *Medial PFC thick.*  Intercept  Linear slope | | - | 0.01  0.01 |
| *Nucleus accumbens*  Intercept  Linear slope | | - | 0.12*  0.06*** |

*Note*. PFC= prefrontal cortex; ACC=anterior

cingulate cortex ; vol. = volume; sa. = surface area;

thick. = thickness; **p<.*05. ***p*<.01. ****p*<.001

Table S4

*Covariances between intercepts and linear slopes
of lateral and medial PFC and in-depth exploration*

|  | Estimate |
| --- | --- |
| *lPFC volume* |  |
| Int lPFC/ACC vol. ↔ Int. in-depth exp | -0.19 |
| LS lPFC/ACC vol ↔ LS. in-depth exp. | 0.02 |
| Int lPFC/ACC vol→ LS. in-depth exp | 0.20 |
| Int in-depth exp → LS LPFC/ACC vol | 0.98*** |
| *lPFC surface area* |  |
| Int lPFC/ACC SA ↔ Int. in-depth exp | -1.68* |
| LS lPFC/ACC SA↔ LS in-depth exp. | -0.07*** |
| Int lPFC/ACC SA→ LS in-depth exp | 0.14*** |
| Int in-depth exp → LS lPFC/ACC SA | 1.95*** |
| *lPFC thickness* |  |
| Int lPFC/ACC Thick ↔ Int in-depth exp | 0.16 |
| LS lPFC/ACC Thick↔ LS in-depth exp. | 0.00 |
| Int lPFC/ACC Thick→ LS in-depth exp | -4,07*** |
| Int in-depth exp → LS lPFC/ACC Thick | -0.04 |
| *mPFC volume* |  |
| Int mPFC vol. ↔ Int. in-depth exp | -0.40 |
| LS mPFC vol ↔ LS in-depth exp. | 0.02 |
| Int mPFC vol→ LS in-depth exp | 1.03*** |
| Int in-depth exp → LS mPFC vol | 0.30* |
| *mPFC surface area* |  |
| Int mPFC sa. ↔ Int in-depth exp | 0.24 |
| LS mPFC sa ↔ LS in-depth exp. | 0.00 |
| Int mPFC sa→ LS in-depth exp | -0.09 |
| Int in-depth exp → LS mPFC sa | -0.57 |
| *mPFC thickness* |  |
| Int mPFC Thick ↔ Int. in-depth exp | -0.08 |
| LS mPFC Thick↔ LS in-depth exp. | 0.01 |
| Int mPFC Thick→ LS in-depth exp | 1.35*** |
| Int in-depth exp → LS mPFC Thick | 0.06 |

*Note*. Int= intercept; LS= Linear slope; lPFC/ACC= lateral

prefrontal cortex/anterior cingluate cortex;

mPFC= medial prefrontal cortex;

Vol. = volume; SA = surface area; Thick. = thickness.

**p<.*05. ***p*<.01. ****p*<.001

Figure S1. Age distribution of the accelerated longitudinal Leiden self-concept study.


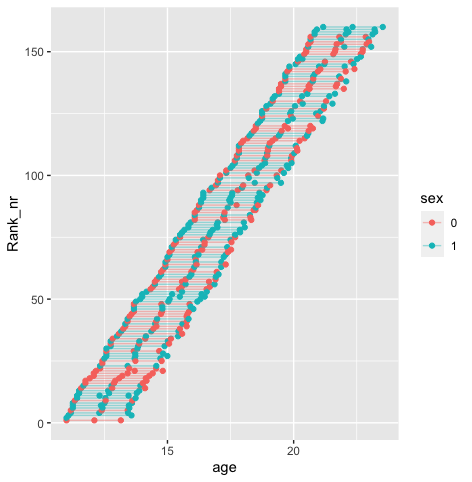

Supplement: Supplementary file 1 [file mmc1.docx]
